# Clinical Evidence of Antidepressant Effects of Insulin and Anti-Hyperglycemic Agents and Implications for the Pathophysiology of Depression—A Literature Review

**DOI:** 10.3390/ijms21186969

**Published:** 2020-09-22

**Authors:** Young Sup Woo, Hyun Kook Lim, Sheng-Min Wang, Won-Myong Bahk

**Affiliations:** Department of Psychiatry, College of Medicine, The Catholic University of Korea, Seoul 07345, Korea; youngwoo@catholic.ac.kr (Y.S.W.); drblues@catholic.ac.kr (H.K.L.); smwang11@naver.com (S.-M.W.)

**Keywords:** clinical trials, insulin resistance, depression, anti-hyperglycemic agents, pathophysiology

## Abstract

Close connections between depression and type 2 diabetes (T2DM) have been suggested by many epidemiological and experimental studies. Disturbances in insulin sensitivity due to the disruption of various molecular pathways cause insulin resistance, which underpins many metabolic disorders, including diabetes, as well as depression. Several anti-hyperglycemic agents have demonstrated antidepressant properties in clinical trials, probably due to their action on brain targets based on the shared pathophysiology of depression and T2DM. In this article, we review reports of clinical trials examining the antidepressant effect of these medications, including insulin, metformin, glucagon like peptide-1 receptor agonists (GLP-1RA), and peroxisome proliferator-activated receptor (PPAR)-γ agonists, and briefly consider possible molecular mechanisms underlying the associations between amelioration of insulin resistance and improvement of depressive symptoms. In doing so, we intend to suggest an integrative perspective for understanding the pathophysiology of depression.

## 1. Introduction

Depression is a complex, heterogeneous illness associated with significant chronicity and considerable impairment of physical ability and psychosocial function [1]. Although antidepressants are the well-established first-line treatment for depression, current pharmacological therapies are often ineffective, yielding high rates of inadequate treatment response, reflected in recurrence or chronicity [2,3]. Furthermore, pharmacological therapy for depression is often associated with intolerance, with clinically substantive side effects including nausea, constipation/diarrhea, dry mouth, somnolence/insomnia, dizziness, sexual dysfunction, weight gain, increased appetite/anorexia, and with uncommon serious adverse effects including prolongation of corrected QT interval, increased risk of fracture, hyponatremia, and inhibition of platelet aggregation [4,5,6]. Therefore, there remains a need to elucidate novel targets that may yield improved efficacy, tolerability and, possibly, disease modifying effects.

The insulin signaling system has been proposed as a novel target in the treatment of depression [7,8,9]. Because a bi-directional association between diabetes/insulin resistance and depression has been described in many studies [9,10,11,12,13], an underlying shared pathophysiologic mechanism of depression and type 2 diabetes mellitus (T2DM) and anti-depressant effects of anti-diabetic agents have been explored in the field. Consequently, a growing body of evidence has suggested that altered insulin signaling, including insulin availability and/or sensitivity or availability of insulin receptors, is important to the underlying pathophysiology of depression [14,15].

Binding of the ligand to insulin receptors (IRs) generates intracellular signals via two main intracellular signaling pathways, the insulin receptor substrate (IRS)-phosphatidylinositol 3-kinase (PI3K)-Serine/threonine kinase (Akt) pathway and the mitogen-activated protein kinase (MAPK)/extracellular signal-regulated kinase (ERK) pathway, which are known to be involved in depression pathophysiology [16,17]. Moreover, insulin signaling may act to regulate mood by modulating neurogenesis. Hippocampal neurogenesis is known to be impaired in both depression and diabetes [18,19]. In addition, recent studies have demonstrated that altered insulin signaling in the brain induces monoaminergic dysfunction [20,21]. Recent pre-clinical and clinical trials have also demonstrated relationships between insulin signaling and depression; knockdown of insulin receptors in the hypothalamus or astrocytes generated depressive behavior in mice [22,23], and insulin administration may have improved mood and cognition in a few human studies as well [14,24,25]. Additionally, several preclinical studies on animal models suggested that high fat diet induced metabolic dysfunction could induce depressive-like behavior [26] via dysregulation of synaptic plasticity and insulin signaling [27], serotonin mediated neurotransmission [28,29], brain indoleamine 2,3-dioxygenase (IDO) activity [30], and intestinal microbiome and brain metabolome [31]. Other trials examining the antidepressant effect of repurposed anti-hyperglycemic (anti-diabetic) agents including the insulin sensitizing agent pioglitazone [32] and glucagon-like peptide-1 (GLP-1) receptor agonists [33,34] also suggest that the attenuation of insulin resistance could be a plausible target for the treatment of depression.

Therefore, the objective of this review was to delineate the antidepressant effects of insulin and anti-hyperglycemic agents in human subjects with depression. In addition, possible pathophysiological explanations for the suggested antidepressant effects of these agents were reviewed.

## 2. Methods

This study is a narrative review investigating the antidepressant effects of insulin and anti-hyperglycemic agents and their implications for the pathophysiology of depression. We searched electronic libraries including PubMed, EMBASE, and PsychINFO for studies published prior to February 2020 applying the filter function for clinical trials based on the following medical subject headings when possible or using text word terms: “Major depressive disorder (MDD)” or “Depression” or “Depressive disorder” or “Depressive symptoms” in combination with “anti-diabetic” or “anti-hyperglycemic” or “insulin” or “insulin receptor sensitizer” or “GLP-1” or “peroxisome proliferator-activated receptor gamma” or “PPARγ” or “metformin” or “liraglutide” or “exenatide” or “pioglitazone” or “thiazolidinedione” or “rosiglitazone”. We included clinical studies with anti-hyperglycemic agents as monotherapy or as add-on treatment for human subjects as well as studies that evaluated depressive symptoms. Articles informed by observational studies and clinical trials, review, or meta-analysis articles relevant to the topic, and other publications cross-referenced for additional published articles were included. Studies were excluded if they included only subjects with diabetes or failed to use human subjects; unpublished data and conference abstracts were also excluded.

## 3. Antidepressant Effects of Anti-Hyperglycemic Agents in Clinical Trials

We found 21 clinical trials that met the inclusion criteria, including four studies of insulin, four of metformin, 11 of PPARγ receptor agonists, and two studies of GLP-1 receptor agonists (GLP-1RA). Table 1 summarizes the main characteristics of these clinical trials.

### 3.1. Insulin

The antidepressant effects of subcutaneous insulin were investigated in two studies with T2DM patients. Reza et al. [35] reported significant improvements in depressive symptoms with subcutaneous insulin treatment in older (aged 65 years and over) type 2 diabetic patients. In this 12-week study, 30 consecutive patients advised to start insulin by the clinical diabetologist were treated with subcutaneous insulin, and 10 consecutive patients who remained on glucose-lowering drugs (combinations of gliclazide, metformin, and acarbose) were recruited as control subjects. Depressive symptoms at baseline, evaluated using the Geriatric Depression Scale (GDS) score, did not differ between the groups. Although the glycemic control remained relatively poor, with only a small reduction in Hemoglobin A1c (HbA1c) during the study in both groups, subcutaneous administration of insulin significantly reduced the GDS scores at both 4 weeks and 12 weeks compared to baseline; there was no change in the control group. However, in a follow-up study [36] of 57 older patients with poorly controlled T2DM, insulin was not associated with any change in depressive symptoms at the end of the 6-month study. The authors compared the effects of continuation of oral medication, change to isophane insulin, and basal/bolus insulin therapy. The results showed that the Hospital Anxiety and Depression Scale (HADS)-B (depression) scores were lower in the basal/bolus insulin group at 1 and 3 months compared to baseline, although the change at 6 months was not significant (*p* = 0.06). Moreover, insulin therapy did not improve depressive symptoms in a meta-analysis of diabetic patients [53]. Rather, diabetic patients on insulin therapy were significantly associated with the higher risk of depressive symptoms.

A few more clinical studies have investigated the effect of intranasal insulin on mood by studying subcutaneous insulin therapy in healthy or non-diabetic subjects. In an 8-week double-blind randomized comparison between intranasal insulin and placebo in 38 healthy subjects [37], intranasal insulin administration improved mood as well as memory. In that study, the authors assessed mood using an adjective check list designed to assess actual mood on 15 dimensions and assessed cognitive function with a word list, word-stem priming, and the Stroop test. After 8 weeks of intranasal insulin, delayed recall was significantly improved, feelings of well-being and self-confidence were increased, and anger ratings were decreased in comparison with placebo. Intranasally administered insulin could attenuate the hypothalamic–pituitary–adrenal (HPA) axis response to psychosocial stress, which has been associated with depression in numerous previous studies [54]. A single intranasal dose of insulin, 5 min before being exposed to the Trier Social Stress Test (TSST), diminished the HPA axis response to the TSST as measured by cortisol taken from saliva and plasma [55]. However, in a double-blind study examining the effect of intranasal insulin administration on cognitive function and mood symptoms in patients with treatment-resistant depression [14], intranasal insulin did not result in statistically significant improvement in overall mood or cognition. No significant difference was found between the effects of intranasal insulin and placebo on changes in total Montgomery Åsberg Depression Rating Scale (MADRS) scores, the Positive or Negative subscales of the Positive and Negative Affect Schedule (PANAS), or a global index of neurocognition.

### 3.2. Metformin

The effect of metformin on mood has been investigated in four studies which included subjects with T2DM and impaired glucose tolerance in polycystic ovarian syndrome (PCOS). In a 1-year diabetes prevention study of subjects with impaired glucose tolerance, Ackermann et al. [38] investigated the association between changes in body weight and changes in health status among three intervention groups (intensive lifestyle intervention, pharmacotherapy with metformin, and placebo) using scores on the Beck Depression Inventory (BDI) as a covariate. BDI total score was significantly, but only slightly, decreased during the 1-year treatment period in all three groups. At 1 year, the changes in BDI total scores were −1.02 in the intensive lifestyle intervention group (*p* < 0.001), −0.71 in the metformin group (*p* < 0.001), and −0.58 in the placebo group (*p* < 0.001). However, it is not clear which intervention led to a greater decrease in BDI scores because the authors did not compare the treatment effects among the three groups. Moreover, as the baseline BDI scores were quite low (3.53 in the intensive lifestyle, 3.84 in the metformin, and 4.05 in the placebo group), the clinical significance of these results may be limited. However, in another randomized clinical trial (RCT), 24-week metformin treatment significantly improved MADRS and 17-item Hamilton Depression Rating Scale (HDRS) scores in patients with depression and T2DM [39]. The reduction in depressive symptoms was paralleled by improvement in HbA1c, but the correlation between HbA1c changes and changes in depressive symptoms was not reported. In that study, chronic treatment with metformin also improved cognitive performance as assessed by the Wechsler Memory Scale—Revised. The authors suggested that administration of metformin may improve depressive symptoms comorbid with T2DM through improvements in cognitive performance. In addition, in a 6-month study of premenopausal women (aged 30–45 years) with T2DM or prediabetes, administration of metformin reduced, albeit insignificantly, the BDI-II score (*p* = 0.088) as well as the number of patients with depressive symptoms (BDI-II score 14 or higher, *p* = 0.056) in women with diabetes but not in those with prediabetes [40].

Metformin was included as an active control in three RCTs. In a study of women with PCOS, 12-week treatment with metformin (−0.3 ± 0.7) was marginally inferior to treatment with myo-inositol (−1.0 ± 1.7) in reducing depressive symptoms as measured by BDI scores [41]. The study’s clinical value in terms of improving depression outcomes is limited because the subjects were not selected for depression, and the baseline BDI scores were relatively low (15.1 ± 3.9 for the metformin group; 15.4 ± 5.2 for the myo-inositol group). Metformin was also inferior to pioglitazone at reducing depressive symptoms in patients with post-stroke depression and T2DM [48] and in patients with concomitant PCOS and MDD [44].

Recently, Moulton and colleagues conducted a meta-analysis of 19 published RCTs (five of metformin, two of peroxisome proliferator-activated receptor (PPAR)-γ receptor agonists, two of incretin-based therapies, and one of insulin) on the effects of diabetes treatments on depressive symptoms. They found that metformin had similar effects to placebo on depressive symptoms (pooled effect size = −0.49, 95% CI = −1.04 to 0.074, *p* = 0.089) and was inferior to active controls (pooled effect size = +1.32, 95% CI = 0.31 to 2.34, *p* < 0.001) [56].

### 3.3. PPARγ Receptor Agonists

Numerous studies have examined the antidepressant effects of PPARγ receptor agonists. PPARγ agonists such as rosiglitazone and pioglitazone have been evaluated in four open-label clinical trials [21,42,45,48] and six RCTs [43,44,46,47,49,51]. Majority of studies were conducted in subjects having metabolic or endocrinologic abnormalities such as T2DM, insulin resistance, obesity, metabolic syndrome (MetS) or PCOS [21,42,44,45,46,48], but some studies investigated antidepressant effects of insulin in subjects without metabolic abnormalities [43,47,49,51].

Rasgon and colleagues [42] tested the hypothesis that the addition of rosiglitazone, an insulin-sensitizing agent, would improve mood in prediabetic patients with unipolar or bipolar depression. The authors additionally administered rosiglitazone with treatment as usual for 12 weeks to eight patients, and the results showed a decline in the mean HDRS score. The improvement in depressive symptoms was not associated with improvements in insulin sensitivity as measured by the Matsuda Index. In open-label studies of another PPARγ receptor agonist, pioglitazone, among subjects with both depressive symptoms and metabolic risk factors, pioglitazone improved depressive symptoms in subjects with MDD and abdominal obesity/MetS [21] and with post-stroke depression and T2DM [48]. Kemp et al. [21] reported that total scores on the Inventory of Depressive Symptomatology (IDS) decreased from 40.3 ± 1.8 at baseline to 19.2 ± 1.8 at week 12 in 23 MDD patients with abdominal obesity or metabolic syndrome patients who received pioglitazone. Moreover, Hu et al. [48] compared the antidepressant effects of pioglitazone versus metformin as adjunctive with fluoxetine in patients with post-stroke depression and T2DM; pioglitazone decreased HDRS scores significantly more than metformin within 3 months of treatment, independently of depression severity.

Furthermore, in two double-blind randomized controlled trials with subjects with depression and comorbid conditions [44,46], pioglitazone showed significant antidepressant effects. Pioglitazone was superior to metformin in reducing HDRS scores at 6 weeks in patients with concomitant PCOS and MDD [44] and to placebo in reducing both depressive and anxiety symptoms, measured using the Hospital Anxiety and Depression Scale (HADS), in nondiabetic MetS patients [46]. Interestingly, the improvement in depressive symptoms was independent of the insulin-sensitizing effects of pioglitazone; in both studies, changes from baseline in homeostatic model assessment of insulin resistance (HOMA-IR) values were not correlated with the changes in depressive symptoms. Meanwhile, Simuni and colleagues [50] reported the results of a phase 2 double-blind RCT of pioglitazone in early Parkinson’s disease, which showed no significant difference between pioglitazone and placebo in changes in GDS scores over 44 weeks.

Two RCTs have been performed in subjects with MDD but without metabolic abnormalities [43,47]. Pioglitazone adjunctive therapy for moderate to severe (17-item HDRS total score ≥22) MDD patients was superior to placebo in reducing the HDRS total score during the course of the 6-week trial [43]. Moreover, the frequency of treatment response, remission (HDRS score ≤ seven), and early improvement (≥20% reduction in HDRS score within the first 2 weeks) was significantly higher in the pioglitazone group than in the placebo group. However, Lin et al. [47] reported no significant difference in the mean decline in HDRS scores between the adjunctive pioglitazone group and the placebo group when subjects both with and without insulin resistance were included. The difference in HDRS score change was only significant in subjects with insulin resistance. Another remarkable finding from this study was the age effect; pioglitazone was more effective in younger patients.

Studies of patients with bipolar depression have also been conducted [45,49,51]. In their proof-of-concept study, Kemp and colleagues [45] reported that adjunctive pioglitazone treatment significantly decreased the total IDS-C30 and Quick Inventory of Depressive Symptoms (QIDS) total scores at the end of an 8-week study in 34 patients with bipolar depression and MetS or insulin resistance. More than three-quarters of these patients, it should be noted, were experiencing treatment-resistant bipolar depression, having already failed two mood stabilizers or the combination of a mood stabilizer and a conventional antidepressant. Additionally, the improvement in depressive symptoms was associated with a reduction in the inflammatory biomarker interleukin (IL)-6. Zeinoddini et al. [49] also reported antidepressant effects of adjunctive pioglitazone relative to lithium in their double blind, placebo-controlled RCT. That study included 48 patients with bipolar I depression and without diabetes or MetS, who had previously failed to respond adequately in the current episode to a trial with lithium plus antidepressant. The total HDRS score was decreased significantly more in the pioglitazone group than in the placebo group over 6 weeks, and the difference was significant onwards from week 2. However, another double-blind placebo-controlled RCT failed to show the antidepressant efficacy of pioglitazone in patients with bipolar depression [51]. Adjunctively administered pioglitazone with concomitant mood stabilizers, antipsychotics, and antidepressants with 38 bipolar depression patients showed significant differences in IDS-C30 and MADRS changes relative to the placebo control group during the 8-week study. However, there was a borderline significant difference between the two groups (*p* = 0.056) in the IDS-C30 score favoring the placebo. The authors noted that the discrepancies between these results and those of previous studies may have stemmed from characteristics of the subjects, including the higher heterogeneity, lower depression severity, and higher concomitant hypomanic/mixed symptomatology of their study population compared with Zeinoddini’s study [49]. In addition, in the pioglitazone group, but not in the placebo group, leptin level was increased in subjects who showed a decrease in depression score, and this correlation was significant [51].

In any case, pioglitazone’s antidepressant efficacy has been supported by meta-analyses [32,56]. In a meta-analysis of four double-blind RCTs [43,44,47,49], pioglitazone induced higher remission rates than control treatments (27% versus 10%, odds ratio (OR) = 3.3, 95% CI = 1.4 to 7.8, *p* = 0.008). Subgroup analysis showed that the OR was even higher in MDD patients (23% versus 8%, OR = 5.9, 95% CI = 1.6 to 22.4, *p* = 0.009) and in the patients without metabolic comorbidities (33% versus 10%, OR = 5.1, 95% CI = 1.5 to 17.9, *p* = 0.01). The reduction in HDRS score was also significant in pioglitazone treatment compared with control treatments (mean difference = 3.3, 95% CI = 2.6 to 4.0, *p* < 0.0001). Another meta-analysis [56], which included four more RCTs [46,48,50,51] than the meta-analysis by Colle et al. [32], also showed a significant treatment effect of pioglitazone (pooled effect size = −0.68, 95% CI = −1.12 to −0.24, *p* = 0.003). Interestingly, being female was significantly associated with improvement in depression, but age, baseline severity of depressive symptoms, baseline HOMA-IR, and baseline fasting glucose were not associated with changes in depressive symptoms.

### 3.4. GLP-1 Receptor Agonists (GLP-1RA)

An open-label, short-term study with liraglutide reported significant reduction in depressive symptoms [33]. In this 4-week study, which included 19 subjects with MDD or bipolar disorder with impaired executive function, administration of liraglutide significantly reduced HDRS scores as a secondary outcome measure, as well as improving executive function. The change in HDRS score did not moderate the improvement in cognition. However, liraglutide failed to show a significant effect on depressive symptoms in an RCT [52]. Treatment with liraglutide for 26 weeks reduced body weight and HbA1c significantly more than standard therapy, but it reduced BDI scores by only one point, which was not significant when compared with the control group.

### 3.5. Safety and Tolerability

The use of insulin could be limited by the risk of hypoglycemia, which is an important adverse effect of insulin therapy. Owing to the risk of hypoglycemia of intravenous insulin infusion, and the fact that high systemic doses would be needed to achieve functionally effective insulin concentrations in the CNS, this mode of administration is not viable for the psychiatric patients [57]. However, intranasal administration allows a non-invasive, direct delivery of insulin to the brain, by bypassing the blood–brain barrier (BBB) and thereby avoiding potential systemic side effects of lowering blood glucose levels [58,59]. In a clinical trial of intranasal insulin, it was well tolerated, and no subject exhibited hypoglycemia or other safety concerns [60].

There are also some adverse reports on the use of metformin. Abdominal pain and other gastrointestinal (GI) symptoms are not uncommon adverse effect of metformin. About 10% of patients stopped taking metformin due to its GI adverse effects [61]. Despite its rarity, lactic acidosis could result in a serious or lethal complication. Furthermore, metformin could be associated with CNS adverse effects. In an animal study, metformin therapy promoted neurodegenerative process by altering tau phosphorylation-dependent pathways in *ApoE* knockout mice [62]. Metformin also impaired cognitive function and increased the risk of Alzheimer’s disease in patients with diabetes [63]. However, there was a contrary report suggesting that metformin use was not related to adverse outcomes of brain structures and cognitive function [64]. These discrepancies suggest that further studies are needed to determine whether metformin truly has a potential role in the prevention or treatment of neurodegenerative disorders [65].

Adverse effects of PPARγ receptor agonists on neuronal function were also noted in both animal and human studies [65]. Rosiglitazone was associated with neuronal apoptosis in the hippocampus of rats [66], and cognitive complications in patients with T2DM [67]. Although the use of PPARγ receptor agonists in T2DM is declining due to its adverse effects, a meta-analysis suggested a relatively favorable safety in MDD patients [32]. There were no deaths, major adverse events, and clinically significant weight gain or weight change [32]. However, caution needs to be exercised because some adverse effects such as increased appetite (15–25%), headache (5–26%), nausea (8.7–25%), sexual dysfunction (20%), abdominal pain (20%), muscular pain (10–17.4%), blurred vision (13–15%), irritability (11.7%), and edema (11.7%) were common after pioglitazone administration [32].

GLP-1RA including liraglutide is commonly associated with GI adverse effects including nausea and vomiting [68]. However, they were all well-tolerated, and no severe adverse events or severe hypoglycemia were reported in an RCT [52]. Moreover, a pooled analysis of phase 2 and 3a trials of liraglutide for weight management showed liraglutide was generally safe with no clinically significant neuropsychiatric safety concerns [69].

## 4. Factors Related to the Antidepressant Effects of Anti-Hyperglycemic Agents

### 4.1. Depression Severity and Class of Anti-Hyperglycemic Agents

Among the aforementioned clinical trials, 11 studies included clinically depressed subjects [14,21,33,39,42,43,44,45,47,49,51]. Among the other studies, some results were positive, and others negative for antidepressant effects of insulin and anti-hyperglycemic agents among subjects who were not diagnosed with depression. Because the efficacy even of antidepressants is not fully supported for subthreshold or mild MDD [70,71], it is not surprising that some of these studies have had negative results. Moreover, the study by Cha et al. [14] was the only study of insulin treatment in MDD patients, but that study also included treatment-resistant subjects who would be likely not to respond well to treatment. In studies with clinically depressed subjects, metformin [39] and liraglutide [33] significantly reduced depressive symptom scores, and six of eight studies of PPARγ receptor agonists reported positive results [21,42,43,44,45,49]. Although a previous meta-analysis of diabetes treatments and their effects on depressive symptoms [56] suggested a significant treatment effect of pioglitazone, but not of metformin, on depressive symptoms, caution is required in interpreting the present results because the studies included here included both clinical (depression) and non-clinical (non-depressed) samples. Furthermore, subgroup analysis of MDD patients in another meta-analysis [32] supported the antidepressant effect of pioglitazone.

### 4.2. Baseline Insulin Resistance

Another point to be clarified is whether the antidepressant effect, if it exists, is demonstrated only in subjects with insulin resistance. Among 11 studies of clinical depression patients, six studies [14,33,43,47,49] included subjects with uncomplicated depression, and five [21,39,42,44,45] included subjects with depression and comorbid T2DM or metabolic abnormalities. Interestingly, all five studies of subjects with comorbid depression and metabolic abnormalities reported positive depression-related outcomes of anti-hyperglycemic agents. Among the six studies of non-diabetic depression patients, antidepressant efficacy was not apparent in three studies [14,47,51]. However, it seems plausible that anti-hyperglycemic agents could have antidepressant effects regardless of the presence of insulin resistance; the effect of baseline insulin resistance on the change in depression severity was not confirmed in studies of subjects with comorbid depression and metabolic abnormalities, which suggests that the antidepressant effect of anti-hyperglycemic agents is not confined to patients with both conditions. Kemp et al. [21] reported that the presence or absence of metabolic syndrome did not appear to affect the change in depression severity. The reductions in depression scale scores were similar in patients with MetS to those in patients without. The effect of baseline HOMA-IR on depression improvement was non-significant in another study [44]. Moreover, a previous meta-analysis [56] reported that baseline HOMA-IR and baseline fasting glucose were not associated with changes in depressive symptoms during pioglitazone treatment. Additionally, a meta-analysis by Colle et al. [32] showed that, in a sub-analysis of patients without metabolic comorbidities, pioglitazone induced greater reductions in HDRS score than control treatment.

### 4.3. Correlation between Changes in Depressive Symptoms and Insulin Resistance

If the improvement in depression is not affected by the presence of baseline insulin resistance, another crucial question would be whether the improvement in depressive symptoms by anti-hyperglycemic agents could be attributable to the amelioration of insulin resistance. In studies of subjects with depression and comorbid T2DM or metabolic abnormalities, Kemp et al. [21] reported a correlation between improvement in depression severity and reduction in insulin resistance. Furthermore, Guo et al. [39] reported that HDRS-17 scores were positively correlated with HbA1c levels and suggested that these findings indicate an association between improvement in depressive symptoms and decreased HbA1c levels by metformin treatment. In a study of subjects with glucose metabolism ranging across the insulin sensitivity spectrum, including insulin-sensitive, insulin-resistant, and/or prediabetic, but not diabetic, subjects [47], HDRS-21 score reduction was significantly associated with changes in oral glucose tolerance and fasting glucose among insulin-resistant patients, but not among insulin-sensitive patients. Additionally, Kashani et al. [44] reported no correlation between changes from baseline in HDRS scores and in HOMA-IR values, but also reported that greater changes in HOMA-IR values predicted greater reduction in HDRS scores, with a near-significant *p*-value (*p* = 0.088). However, no correlation between improvement in depressive symptoms and changes in metabolic parameters was observed in a study by Rasgon et al. [42], which included depressed patients with insulin resistance, and in another study by Aftab et al. [51], which included patients with bipolar depression and varying levels of metabolic abnormalities. To summarize, although most studies supported an association between changes in depressive symptom severity and insulin resistance and/or glucose homeostasis, whether anti-hyperglycemic agents have antidepressant effects through reduction in insulin resistance remains uncertain.

## 5. Pathophysiologic Implications of Clinical Trials

### 5.1. Proposed Mechanism Underlying Links between Depression and Diabetes

A body of evidence has addressed the correlation between depression and T2D. Although evidence from epidemiological studies strongly suggests reciprocal associations between the two conditions [13,72,73], various confounding factors implicated in their comorbidity complicate the association [7]. However, several possible molecular links between depression and diabetes have been identified.

#### 5.1.1. Brain Insulin Resistance

Brain insulin resistance is suggested as one of the most important mechanisms linking the pathophysiology of diabetes and depression. Insulin signaling pathways are triggered by the binding of ligands such as insulin-like growth factor (IGF) 1 and 2 and insulin to the transmembrane IR, and it induces several downstream signaling events involving different insulin-induced kinases such as PKC, AKT, mTOR, S6K1, SIK2, extracellular signal-regulated kinase 1/2 (ERK1/2), and ROCK1 [74,75]. The IR activities are distinct from metabolic IR functions in peripheral tissues, which highlight region specificity of IR properties. There are two IR isoforms, A and B, and the two IR isoforms have distinct structures and functions [76]. IR-A is a short isoform found solely in brain, and IR-B is a long isoform predominantly distributed in peripheral metabolic organs such as muscle, liver, kidney, and fat tissue. In brain, both IR-A and -B isoforms are abundantly expressed in olfactory bulb, hypothalamus, hippocampus, cerebral cortex, and cerebellum. However, on a cellular level, neurons express exclusively the IR-A, while astrocytes express both IR-A and IR-B [76]. IR-A also has 5–10 times higher binding affinity for IGF-2 than IR-B, and unlike IR-B, it binds IGF-2 even under physiologically relevant concentration and results in receptor activation [76]. Dysregulation of neuronal plasticity is known be associated with neuropathologic processes, and recent study studies suggest that IGF-2 might play as an essential regulator of neuronal plasticity [77,78,79]. Therefore, the IGF-2 sensitive, neuron-specific pattern of IR-A may underlie functions of IR that are characteristic for the brain [76].

Deficiencies in IR activation, insulin availability, and downstream IR-related mechanisms may result in aberrant IR-mediated functions and, subsequently, a broad range of brain disorders, including depression. In preclinical studies, depressive-like behaviors were triggered by insulin receptor knockout in the hypothalamus [22]. The association between impaired brain insulin signaling and depressive-like behavior could be mediated by mitochondrial dysfunction, increased reactive oxygen species, or leptin signaling, as suggested by studies with neuronal-specific knockout of insulin receptor (NIRKO) mice [15,80].

Furthermore, recent evidences suggested that cross-talk between insulin and glutamate signaling in excitotoxicity may play important roles in the brain insulin resistance [81]. Insulin modulates currents of the *N*-methyl-d-aspartate (NMDA) receptor in a dose-, time-, and NMDA subunit-specific manner through the IGF-1 receptor signaling pathway [82,83] and increases the number of functional NMDA receptors in the cell membrane [84,85]. It has been reported that insulin increases the vulnerability of rat cortical neurons to the excitotoxic effects of glutamate, thereby contributing to cell death [86,87]. However, Nampoothiri et al. [88] reported conflicting results, in which they demonstrated that insulin blocks glutamate-induced neurotoxicity, thereby contributing to cell survival. The discrepancy among these results may be due to differences in experimental conditions. Moreover, as suggested by the result from Datusalia et al. [89] which showed that long-lasting hyper-insulinemia increases glutamate excitotoxicity in cortical neurons of insulin-resistant rats, the development of insulin resistance, caused by prolonged insulin exposure, may mask insulin effects [85]. Concurrently, a recent study showed that the short-term insulin exposure can improve neuroprotection against excitotoxicity and prevent glutamate mediated excitotoxicity whereas chronic insulin exposure results in neuronal insulin resistance and worsen excitotoxicity [85]. Although the exact molecular link between insulin resistance and glutamate excitotoxicity remains elusive, it has been suggested that the excitotoxic glutamate may induce the development of neuronal insulin resistance via inhibiting the IR/Akt/mTOR pathways [81]. As noted in previous studies, an abnormal increase in intracellular free Ca^2+^ concentration ([Ca^2+^]i) induces collapse of mitochondrial potential and result in the glutamate-induced excitotoxicity [90]. Pomytkin et al. [81] reported that activation of IR, Akt and mTOR, and inhibition of glycogen synthase kinase (GSK)3β were significantly decreased when significant mitochondrial depolarizations occurred due to glutamate-evoked massive influxes of Ca^2+^ into the cells.

It has been also suggested that elevated branched chain amino acids (BCAA) plasma levels may play a role in the link between depression and T2DM. In an animal model, chronic treatment with metformin normalized glucose metabolic impairments, suggesting that insulin resistance accompanied by elevated circulating levels of BCAAs in male mice fed a high-fat diet, which is in parallel with improvement in high fat diet-induced depressive-like effects [91]. Given that the elevated levels of circulating BCAA predict the severity of insulin resistance [92] while the rate of 5-hydroxytryptamine (5-HT) production may be limited because the uptake of tryptophan into the brain through BBB by the large neutral amino acid transporter 1 (LAT1) compete with BCAA [93], the rate of serotonin synthesis may be affected by BCAA [94]. In their study, Zemdegs et al. [91] suggested that metformin may produce antidepressant-like effect by decreasing circulating BCAA levels to favor serotonergic neurotransmission in the hippocampus.

Moreover, impaired insulin signaling may contribute to the pathogenesis of depression by regulating dopaminergic neurotransmission. Increased monoamine oxidase expression and dopamine turnover were observed in NIRKO mice [15], and decreased dopamine release was observed in astrocyte insulin receptor knockout mice [23]. Cai and colleagues [23] suggested that the association between the dopamine system and insulin signaling could be moderated by reductions in purinergic signaling on dopaminergic neurons. In addition, alterations in dopamine neurotransmission in the reward circuitry were also disrupted by a high-fat diet, inducing depressive-like behavior in mice through mediation of impaired brain insulin signaling [26]. The suggested mechanisms that mediate insulin signaling and dopaminergic neurotransmission, including mitochondrial dysfunction [95,96,97], oxidative stress [98,99,100], impaired leptin [101,102] and purinergic signaling [103], and disrupted dopaminergic neurotransmission [104], are known to play important roles in the pathophysiology of depression.

#### 5.1.2. Immune/Inflammatory, Oxidative, and Nitrosative Stress

A body of the literature suggests that activated immune/inflammatory and oxidative stress (IO and NS) pathways and consequent mitochondrial dysfunction may play a role in the pathophysiology of major psychiatric disorders including MDD by causing dysfunction in synaptic or neuronal plasticity and neuronal signaling, as well as in neuronal survival and death [105,106,107,108]. IO and NS pathways are also thought to contribute to the pathways underpinning the development of metabolic disorders, including insulin resistance [109].

It is well known that some pro-inflammatory biomarkers, including interleukin (IL)-6, tumor necrosis factor (TNF)-α, and C-reactive protein (CRP), are commonly increased in diabetes [110] and depression [111], and evidence indicates that depression and T2DM share biological mechanisms, namely, overactivation of the immune response, leading to a cytokine-mediated inflammatory response [112]. TNF-α is one of the most thoroughly studied pro-inflammatory biomarkers for both depression and T2DM. In preclinical studies, TNF-α administrated by an intracerebroventricular route produced depressive-like behavior, which was counteracted by anti TNF-α antibody and knockout of the TNF-α receptor 1 [113]. The depressogenic effect of TNF-α may be related to impaired serotonergic neurotransmission. TNF-α can reduce central nervous system (CNS) serotonin levels by increasing the degradation of tryptophan through induction of IDO, an enzyme that catabolizes the serotonin precursor tryptophan in a TNF-α dependent manner and activating the serotonin transporter (SERT) [114,115]. Furthermore, TNF-α can impair insulin signaling. TNF-α activates stress-response kinase pathways, including the IκB kinase β (IKKβ)/nuclear factor κ B (NFκB) and c-Jun *N*-terminal kinase (JNK) pathways, which promote insulin resistance via phosphorylation of IRS1 on serine residues [116], and by decreasing expression of GLUT-4 glucose transporters, which are crucial to insulin transmembrane signaling [75,117]. In addition, TNF-α was shown to disrupt blood–brain barrier (BBB) integrity in a mouse model of depression [118] and in patients with T2DM [119], and to increase BBB permeability, which is restored by treatment with anti-inflammatory drugs [118]. Increased BBB permeability could allow for easier entry of both cytokines and immune cells into the brain [120], exacerbating the allostatic load to the HPA axis, leading to its dysregulation [7].

Disturbed antioxidant defenses in depression are indicated by reduced antioxidant molecules, including ubiquinone (coenzyme Q10), vitamins D and E, high-density lipoprotein cholesterol (HDL-c), glutathione peroxidase, and zinc [106,121,122]. The product of lipid peroxidation, malondialdehyde (MDA) [123,124] and activities of antioxidative enzymes such as catalase (CAT) and superoxide dismutase (SOD), along with reduced glutathione (GSH), also indicate the role of oxidative stress in depression [124]. In addition, plasma activity of Paraoxonase 1 (PON1), a potent antioxidant that protects against lipid peroxidation, is lower in patients with depression [125]. The imbalance between oxidative stress and antioxidative defenses leads to dysregulation of brain functions and abnormalities in neuronal signaling, which have been shown to be correlated with the pathogenesis and progression of depression [126]. Phospholipids in the brain are susceptible to reactive oxygen species (ROS)-mediated peroxidation. Increased peroxidation of lipids in the brain is an important event in the pathogenesis of depression [127,128]. Depression is also accompanied by nitrosative and nitro-oxidative stress. Nitric oxide (NO), which is produced by NO synthase (NOS), plays a critical role as a neurotransmitter in the CNS, although high levels are associated with neuronal damage and apoptosis [129]. In depression, the activity of endothelial or neuronal NOS was reduced, but plasma levels of nitrate and nitrite levels were increased and normalized after recovery from depression [130]. Increased levels of serum NO accompany depressive symptoms such as psychomotor retardation, sexual dysfunction, weight loss, fatigue, irritability, cognitive dysfunction, and increased suicidal risk [130,131,132]. This NO dysregulation in depression may be the consequence of increased production of NO due to increased production of inducible NOS (iNOS), a main NOS isoform in the inflammatory cells, including glial cells capable of producing higher levels of NO [133,134].

Oxidative and nitrosative stress pathways also play a central role in metabolic disorders including insulin resistance [135]. Increased free radicals, together with reduced antioxidants in adipocytes, have been reported in numerous studies [75]. For example, the activity of PON1 is lowered, and the levels of MDA are increased in people with MetS [109]. The imbalance of oxidative stress and antioxidant defenses can potentially reduce insulin sensitivity and increase insulin resistance in peripheral tissues [109]. It is suggested that insulin resistance can be induced by oxidative stress, which impairs insulin signaling. Oxidative stress activates serine/threonine kinase cascades, such as IKKβ/NFκB and JNK, which, in turn, promote insulin resistance via phosphorylation of IRS proteins, accelerating the degradation of IRS [136] and decreasing expression of GLUT-4 glucose transporters that are crucial to insulin transmembrane signaling [75,117]. Interestingly, patients with insulin resistance have high MDA levels, and MDA concentration is associated with waist-to-hip ratio [137], suggesting a possible contributory role of obesity in the development of insulin resistance via IO and NS pathways. In obese subjects, tissue expression and circulating concentration of pro-inflammatory cytokines and adipokines have been observed [138]. Furthermore, PPARγ, the master regulator of adipogenesis and adipocyte differentiation, is downregulated or dysregulated in obese patients [139]. Systemic oxidative stress upregulates stress-activated kinases (SAPK) including JNK and p38 MAPK, and SAPK phosphorylates the serine residues on PPAR to loss of activity. Hence, elevated levels of ROS and RNS could explain PPAR downregulation in obesity [107]. Moreover, NAD(+)-dependent histone deacetylase sirtuin-1 (SIRT1) expression is reduced in obesity, at least in part via iNOS-induced inactivation of SIRT1 [140,141]. Deficiency of SIRT1 in obesity leads to elevated inflammation characterized by increased mRNA expression of NFκB and pro-inflammatory cytokines in white adipose tissue [142].

In summary, activated IO and NS pathways are implicated in the pathogenesis of depression and metabolic comorbidities including insulin resistance. These metabolic disturbances activate IO and NS pathways and exacerbate dysfunctions in neuroplasticity, synaptic and neuronal signaling, apoptosis, and cell death in many psychiatric disorders. In the process, pro-inflammatory cytokines and oxidative/nitrosative stress, PPARγ inactivation, and SIRT1 deficiency are considered to be involved [107].

#### 5.1.3. Neurogenesis and Neuroplasticity

Another possible shared mechanism that may cause both depression and diabetes is defective neurogenesis and synaptic plasticity. Considerable evidence suggests that insulin positively affects hippocampal synaptic and structural plasticity. Presynaptically, insulin increases basal neurotransmitter release and the density of dendritic spines [143]. Moreover, insulin promotes synaptic plasticity by modulating long-term potentiation (LTP) or long-term depression (LTD) at hippocampal synapses through a metaplastic mechanism [144]. The postsynaptic effects of insulin are mediated by PI3K activation [144] and increased membrane recruitment of *N*-methyl-d-aspartate (NMDA) receptors [84]. Insulin modulates glutamate receptor activity by enhancing phosphorylation of both NR2A and NR2B subunits of NMDA receptor [83] and clathrin-dependent endocytosis of the GluA1 subunit of a-amino-3-hydroxy-5-methyl-4-isoxazolepropionic acid (AMPA) receptors [145]. Downregulation of AMPA receptor activity in excitatory synapses of hippocampal CA1 neurons is fundamental for insulin-induced LTD [146]. Moreover, insulin may impinge on structural features of synapses, as revealed through enhanced dendritic spine formation by IRSp53 [147] and increased basal dendrites of CA1 pyramidal neurons by insulin-like growth factor (IGF)-1 stimulation [148]. Accordingly, mice rendered serum IGF-1 deficient by knocking out IGF1 show reduced density of glutamatergic synapses and disrupted LTP [149]. In addition, astrocytes, which play essential roles in synaptic plasticity, are also insulin responsive [150]. Furthermore, lack of proper central insulin signaling would result in dysfunctional hippocampal neurogenesis, which may play a role in the pathophysiology of depression and T2DM [7,151]. Energy metabolic and nutritional cues have been suggested as major physiological determinants of neural stem cell (NSC) fate and modulators of the whole neurogenic process. Insulin/IGF-1 cascade, mitochondrial activity, the 5′ AMP-activated protein kinase (AMPK)/mTOR axis, and the transcription regulators CREB and SIRT1 are well represented in the molecular networks that dictate neural-stem-cell self-renewal, migration, and differentiation [152]. Indeed, insulin may exert either trophic or harmful effects on NSCs depending on the timing and the duration of stimulation. Insulin and IGF-1 promote neurogenesis by modulating NSC proliferation, differentiation, and survival, but chronic hyper-activation of insulin/IGF-1 signaling cascades can cause premature depletion of the NSC reservoir [153,154,155]. Moreover, calorie restriction, which reduces plasma levels of glucose and insulin, enhances neurogenesis in the dentate gyrus, induces expression of brain-derived neurotrophic factor (BDNF), and regulates proliferation and self-renewal of NSCs and controls their reservoir by cell-autonomous mechanisms involving metabolic sensors such as SIRT1 and CREB in the hippocampus [156,157]. Conversely, ablation of the expression of genes encoding nuclear transcription factor forkhead box O transcription factor (FOXO), which mediate the inhibitory action of insulin or IGF-1 on neurogenesis and survival, induces premature neuronal differentiation and rapid depletion of NSCs [158] and aberrantly elevates the nutrient-dependent mTOR pathway, reducing self-renewal and accelerating premature differentiation of NSC [159]. Together, these findings emphasize the essential roles played by insulin in regulating neurogenesis, neuronal development, and synaptic plasticity.

#### 5.1.4. Hypothalamic–Pituitary–Adrenal Axis

Another point that should be addressed is the allostatic load and the HPA axis, a key component mediating chronic psychological stress and depression or T2DM. Variations in the glucocorticoid (GC) level have been repeatedly reported in the blood of patients with depressive symptoms and T2DM, as compared to healthy controls [160,161]. Moreover, HPA axis dysregulation is among the most reliable of biological findings in depression and is implicated in the development of insulin resistance. However, the correlations of stress with diabetes and the metabolic effects of GC on the brain are still poorly understood [162,163]. GCs are major regulators of energy metabolism, playing key roles in the counter-regulatory responses and metabolic adaptations to the increased energy demand provoked by stress. In general, GCs mobilize energy substrates such as lipids and glucose, increasing their levels in systemic circulation [164]. Physiologically, GCs promote insulin secretion [165]; conversely, insulin activates the HPA axis and leads to an increase in GC levels and corticotropin releasing hormone (CRH) levels [166]. Increases in CRH by insulin are mediated through activation of the cyclic adenosine monophosphate (cAMP)/AMP protein kinase A (PKA) and PI3K/AKT pathways; this activation, in turn, affects CRH promoters, stimulates CRH gene transcription, and increases CRH levels [166].

Under chronic stress conditions, GC receptor resistance develops and disrupts HPA axis regulation [167]. The subsequent uninhibited release of GCs triggers physiological cascades that play a role in the pathophysiology of metabolic and psychiatric pathology, including insulin resistance and obesity [168]. In preclinical studies, chronic stress or long-term GC exposure led to depressive-like behavior as well as insulin resistance in rats, possibly mediated by the cAMP/CREB pathway and insulin signaling involving the signal transducer and activator of transcription 3 (STAT3) and PI3K-AKT-FOXO1 pathways damaged by inhibiting the activation of IRS2 and PI3K [169,170]. Moreover, administration of exogenous GC can induce an insulin-resistant brain state by altering gene expression of insulin [171]. Chronic GC exposure also can induce increased expression of JNK in the hippocampus, which is associated with insulin receptor-mediated inactivation of the Akt/glycogen synthase kinase (GSK) 3β pathway, which leads to depressive symptoms [172]. While long-term GC exposure induces insulin resistance, insulin resistance can induce glucocorticoid receptor insensitivity and HPA axis dysregulation. One of the mechanisms by which insulin affects the HPA axis is in its effect on blood glucose [173]. When insulin does not effectively lower blood glucose, its effect on the HPA axis is diminished [166]. These results suggest that the bidirectional physiological link between depression and diabetes is likely to involve the HPA axis.

#### 5.1.5. Gastrointestinal Microbiome

The accumulating knowledge suggests that disruptions to the gut microbiome can contribute to both metabolic and depressive disorders. Recent evidences indicate that gut microbes and insulin interact with each other. Reducing insulin resistance could maintain the homeostasis of gut microbiota and restore intestinal microbiome disorders [166]. Inversely, modulation of gut microbiota could improve glucose metabolism and insulin resistance by adjusting the microbiota–gut–brain axis [174]. Probiotics can modulate the HPA axis, inflammation, and neurotransmitters [175,176]. Indeed, gut microbiota modification can reduce depressive behavior through enhancing brain insulin signaling, decreasing inflammation, and modulating neurotransmitters and other neuroactive molecules such as BDNF, GABA, and tryptophan in affect-related brain structures including amygdala and nucleus accumbens [177,178]. Moreover, insulin sensitizing agents probably have both direct and indirect effects on gut bacteria. Studies in rodents and humans tend to agree that metformin treatment increases the relative abundance of bacteria which produce short chain fatty acid and stimulate the secretion of GLP-1 and GLP-2, and thereby increasing insulin and adiponectin expression, contributing to enhanced insulin sensitivity [179,180]. The relative abundance of genus *Akkermansia*, mucin-degrading bacteria, increases in metformin-treated mice on a high-fat diet (HFD). This positive influence is probably due to metformin action on mucin-producing goblet cells in the intestine which underlie the improved glucose profiles associated with *Akkermansia* [181]. GLP-1RA including liraglutide can also alter gut microbiota. Substantial rearrangement of the bacterial structure was observed in mice treated with liraglutide [182]. The authors postulated that GLP-1RA delays the gut transit time and gastric emptying rate, and it could modify the gut lumen internal environment and thus affect microbiota composition. Additionally, this may be because PPARγ agonists have anti-bacterial activity, and they may indirectly affect gut bacteria [183]. Bai et al. [184] showed that pioglitazone administration in high fat-fed rats reduces the abundance of proteobacteria, which seems to correlate with the decrease in pro-inflammatory cytokines and plasma endotoxin levels. In summary, the main effect of insulin sensitizing agents is thought to be on the microbiome composition, basically increasing the short chain fatty acid-producing bacteria responsible for losing weight and suppressing inflammation [180].

### 5.2. Linking Clinical Evidence and Pathophysiology: Possible Mechanisms of Antidepressant Effects of Anti-Hyperglycemic Agents

As noted above, to date, insulin-based medications have shown disappointing results. The lack of significant antidepressant effect from insulin-based medications suggests that insulin resistance may disrupt the first steps of insulin signaling by the insulin receptor, and that approaches that bypass the first steps of insulin signaling might show better results [7]. Clinical trials and meta-analyses support the antidepressant effect of PPARγ receptor agonists, and studies with metformin and liraglutide have yielded discrepant results.

Liraglutide is an incretin analog that acts as an agonist to GLP-1 receptor. GLP-1, which is an endogenous incretin hormone released after food intake, is mainly secreted by intestinal L cells, but also by other organs including the CNS. The receptors of GLP-1 are expressed in the pancreas and peripheral tissues including the heart, kidney, lung, and gastrointestinal tract, as well as various brain regions such as the brainstem, arcuate nucleus, area postrema, hypothalamus, nucleus tractus, and vagal afferents [185,186]. Activation of GLP-1 receptor signaling promotes the facilitation of glucose utilization through glucose-induced insulin release and suppression of glucagon release from the pancreas [33]. It is also well established that GLP-1 acts as a neuropeptide that provides glycemic homeostasis [187] and appetite/food intake control [188]. GLP-1, as well as its analogues, can readily cross the BBB and exert various physiological effects [189]. GLP-1 is also able to facilitate glucose transport across the BBB, which is crucial in the homeostasis of brain metabolism [190]. Additionally, growing evidence shows that GLP-1 modulates inflammation and has neuroprotective and neurotrophic effects whose relationship to the pathophysiology of depression is widely accepted [191,192,193]. The important downstream signaling pathway activated by the GLP-1 receptor is the Akt pathway [194], which has the ability to phosphorylate proteins regulating neurogenesis, neuronal development, and synaptic plasticity such as GSK3β, FOXO1, and mTOR (see Section 5.1.3). Moreover, it is reported that GLP-1 signaling regulates dopamine release in striatal neurons [195,196]. As central insulin and dopamine action are interlinked and have impacts on mood, reward, and food intake [197], the dopaminergic system could become compromised under brain insulin resistance. Indeed, in animal models of neurodegenerative disease, GLP-1RAs were able to protect dopaminergic neurons and improve neuropathological features and cognitive functions [198].

However, in an RCT, liraglutide did not improve depressive symptoms despite improvement in metabolic parameters [52]. Moreover, although an open-label study reported significant improvements in HDRS scores and cognitive performance by adjuvant liraglutide administration in subjects with mood disorder [33], the antidepressant effects are not clear because that study included subjects with cognitive impairment at baseline (below average performance in the Trail Making Test-B) and mild depressive symptoms (mean HDRS score = 12.6). One possibility that should not be overlooked is the moderating role of cognitive impairment in the effect of liraglutide on depressive symptoms. Cognitive dysfunction is known to be a psychopathological domain that is relevant in both diabetes and depression. Furthermore, GLP-1RA, including liraglutide, have pro-cognitive effects via molecular pathways and contribute to the pathophysiology of depression and to antidepressant efficacy; GLP-1RA has direct roles in the neurogenesis of the dentate gyrus [199], beneficial actions on mitochondria via the PI3K/Akt pathway and upregulation of antiapoptotic proteins such as Bcl-2, and neuroprotective effects by decreasing neuroinflammation and improving control of synaptic plasticity [194]. However, although different therapeutic strategies including liraglutide can show pro-cognitive effects in depressed patients, research to date has not been designed to distinguish direct pro-cognitive effects from indirect effects on cognition via improvements in mood [200,201]. Given that cognitive improvement was positively correlated with baseline metabolic disturbance, including insulin resistance and body mass index (BMI) in a previous study [33], it is possible that certain subpopulations in whom the dominant psychopathology of depression takes the form of cognitive deficits with underlying metabolic impairment would be more responsive to an intervention that targets glucose and insulin metabolism [202].

Metformin is currently the first-line pharmacological agent for the management of T2DM. Although the mechanisms by which it improves insulin sensitivity are not completely understood, metformin has traditionally been thought to act on the liver to control blood glucose levels by reducing hepatic glucose production and enhancing insulin sensitivity through several molecular pathways including mitochondrial control and AMPK activation [203]. Metformin could also affect glucose metabolism via actions on the intestines such as increasing glucose utilization by the gut, increasing GLP-1 secretion, altering the intestinal microbiome, and suppressing hepatic glucose production via the nucleus tractus solitarius and vagal efferents through AMPK and GLP-1 receptor activation [203]. In the CNS, metformin exercises its effects through many molecular pathways and mechanisms, although conflicting results have been found. Experimentally, metformin has been shown to act as an antidepressant, at least in part, by regulating AMPK signaling, a key enzyme for cellular energy homeostasis. Moreover, a decrease in phosphorylated AMPK (pAMPK) is closely associated with depression-like behaviors in mice under chronic stress [204]. Metformin inhibits mitochondrial respiratory chain complex I, thereby increasing the AMP/ATP ratio, and this lack of energy leads to activation of AMPK. Metformin can also activate AMPK by accumulation of reactive nitrogen species, in turn stimulating the c-Src/PI3K pathway and generating molecules inside the cell to promote AMPK activation [205]. Previous research has suggested that AMPK activation can increase the expression of BDNF, which plays a vital role in the maintenance of synaptic plasticity and depression/anti-depression by activating CREB and Akt/GSK3 signaling pathways [206] and mTOR signaling [207], and by regulating DNA hydroxymethylation via the AMPK/Tet2 pathway [204].

Metformin also has antioxidant properties and regulatory effects on immune response. Although it is not clear whether metformin is able to cross the BBB to reach the brain and exert a direct effect on the CNS, it seems that metformin may cross the BBB and play a central role as Lv et al. reported [208]. As mentioned above, metformin inhibits mitochondrial respiratory chain complex I, which reduces ROS levels, and reduces the production of NO, prostaglandin E2 (PGE2), and pro-inflammatory cytokines (IL-1β, IL-6, and TNF-α) [209]. As compelling research has indicated a role for systemic inflammation and oxidative stress in the pathophysiology of MDD [124,210,211], better understanding of the possible antidepressant effects of metformin would provide insights into the pathophysiology of depression. One of the key factors determining the antioxidative effect of metformin is nuclear factor erythroid 2-related factor 2 (Nrf2). Metformin activates Nrf2, a transcription factor that regulates the expression of multiple antioxidant genes, in an AMPK-dependent manner [212]. The imbalance between ROS and antioxidative defenses leads to dysregulation of brain functions and abnormalities in neuronal signaling, and these have been shown to be correlated with the pathogenesis and progression of depression [126]. Several studies have reported that metformin attenuates elevated MDA levels and restores levels of GSH in states of imbalanced oxidation [212]. Moreover, a notable number of studies have demonstrated a role for Nrf2 as a key element in modulation of inflammatory and cell-mediated immune processes. In the brain, Nrf2 is a key regulator in reducing inflammatory damage, and the absence of Nrf2 may induce more aggressive inflammation through activation of the NFκB pathway [213]. Indeed, some studies have identified possible antidepressant effects of Nrf2 activators [214]. Additionally, as described above, the antidepressant-like effect of metformin may be associated with serotonin system and its projection to hippocampus [91]. Furthermore, metformin can decrease the expression of pro-inflammatory cytokines including IL-1β and IL-6 regardless of diabetes status [215,216] and reduce levels of pro-inflammatory cytokines which result in increased bioavailability of serotonin through regulation of multiple pathways including the tryptophan/kynurenine system [217,218,219]. In summary, the possible antidepressant effect of metformin supports the involvement of inflammation and oxidative stress in depression via diverse signaling molecules and pathways including Nrf2, pro-inflammatory cytokines and the AMPK/BDNF and NFκB pathways. The mechanisms of action of metformin on depression need further validation.

PPARγ agonists, also named thiazolidinediones, have been relatively well investigated and supported as potential candidates for the treatment of depression. PPARγ agonists have insulin sensitizing properties and are commonly used for the treatment of T2DM and MetS. These agents are potent activators of PPARγ, one of three subtypes of PPARs (others are PPARα and PPARβ/δ), which are members of the nuclear receptor superfamily [220]. Upon ligand binding, PPARs translocate into the nucleus and regulate gene expression in a variety of processes including lipid and glucose metabolism, inflammation, atherosclerotic plaque formation, vascular tone, angiogenesis, cellular differentiation, myelinogenesis, glial cell maturation, and fertility [221]. PPARγ receptors are highly expressed in adipose tissue and regulate genes that control cellular energy homeostasis (see Section 5.1.2); their activation is engaged in multiple roles including differentiation of adipocytes, expression of mitochondrial uncoupling proteins, downregulation of leptin, and interaction with multiple genes that control insulin sensitivity [221,222]. Accordingly, PPARγ agonists have been shown to decrease visceral fat, reduce inflammation, and increase insulin sensitivity [223,224]. Moreover, PPARγ agonists are also able to cross the BBB effectively, exerting anti-inflammatory properties and improving insulin resistance in the brain [58]. In addition, the PPARγ agonists have been shown to improve insulin receptor signaling dysfunction by increasing neuronal insulin-stimulated Akt/PKB activation and to improve insulin-induced synaptic plasticity and reverse mitochondrial dysfunction during mitochondrial oxidative stress [21,225]. The antidepressant-like effect of PPARγ agonists may be mediated by monoaminergic neurotransmission. An animal study showed that treating obese animals with PPARγ agonists ameliorated depressive-like behavior [226]. This study used a diabetic mouse strain (db/db) which harbors an autosomal recessive point mutation in gene encoding for the long isoform of the leptin receptor. db/db mice mimic clinical type-2 diabetic conditions such as obesity, hyperglycemia, hyperinsulinemia, hyperphagia, polydypsia, polyurea, impotency, pancreatic β-cell hyperplasia and hypertrophy [226,227], and they also show impairments in dopamine metabolism in the amygdala [228]. The results from this study indicate that insulin action improves mood via modulation of dopamine signaling. Additionally, the literatures [229,230,231] suggested that PPAR-γ activity could be stimulated by serotonergic neurotransmission. Indeed, in fat cells, serotonin leads to the activation of PPAR-γ responsive genes and enhances lipid accumulation [32]. Consequently, the association between antidepressant effect and the activity of the PPAR-γ pathway could be modulated by serotonin system functioning. Recently, Hegazy et al. [232] reported that high fat diet induced obesity was associated with reduced catechol O-methyltransferase (COMT) expression with a concomitant increase in plasma catecholamines as well as metabolic effects such as glucose intolerance, derangement of the lipid profile, and increased systolic blood pressure. Pioglitazone treatment ameliorated the HFD-induced metabolic changes and up-regulated COMT expression with a subsequent reduction in plasma catecholamines levels.

Research addressing whether the beneficial effect of PPARγ agonists on depressive symptoms is correlated with changes in insulin resistance level has been equivocal. Some studies have found no correlation [42,46,51], whereas others have reported marginal [44] or positive results [21]. Alternatively, pathways other than insulin resistance may mediate the antidepressant properties of PPARγ agonists. The improvement in depression correlated with normalization of pro-inflammatory biomarkers, including pro-inflammatory adipokines (leptin) or cytokine (IL-6), suggests a role for inflammatory pathways and intriguing links between PPARγ activation, depression, and inflammation [45,51].

## 6. Conclusions

Disappointingly, evidence suggest that treatment of anti-hyperglycemic agents to depression have shown limited success. Although the antidepressant effects of PPARγ receptor agonists were supported in multiple clinical trials and meta-analyses, the antidepressant efficacy of anti-hyperglycemic agents so far has been equivocal. Since a bidirectional relationship between insulin resistance and depression is supported by several bodies of evidence, it is important to clarify the potential association of these two conditions and explore their common pathophysiological basis. Evidence from investigations of the antidepressant properties of repurposed anti-hyperglycemic agents may provide new insight into the role of brain insulin signaling in the pathophysiology of depression. In the present article, we reviewed the antidepressant properties of anti-hyperglycemic agents including metformin, PPARγ receptor agonists, and GLP-1RA and addressed the underlying mechanisms of these properties. However, although clinical trials have offered some encouraging results, few studies have addressed a sufficient number of biomarkers to enhance our understanding of the molecular and cellular mechanisms involved in the pathophysiology of depression and the antidepressant properties of anti-hyperglycemic agents. Additionally, other important issues are not addressed in this review. Insulin-sensitizing agents may improve the response to antidepressant drugs. Differential impact of changes in central versus peripheral insulin signaling system during pathological conditions and pharmacotherapy with agents targeting insulin receptor should also considered to gain a better understanding of the antidepressant effects of anti-hyperglycemic agents. Unraveling the relationships between depression and impaired insulin signaling could be a matter of great importance as we pursue an understanding of the associated pathophysiology and explore alternative treatment options. Further studies focusing on this issue are warranted.

## Figures and Tables

**Table 1:** Characteristics of prior studies investigating antidepressant effects of anti-hyperglycemic agents.

Author (Year).	Study Design	Subjects	Psychiatric Diagnosis	Number of Subjects	Intervention		Control		Study Duration	Depressive Symptom Measure	Results	Correlations of Depression with Glucose Intolerance or Insulin Resistance
					Drug	Dose	Drug	Dose				
Reza et al. [35]	UB, controlled	T2DM, older subjects (≥65 years)	Absence	40	Insulin (SC), (*n* = 30)		Oral therapy (*n* = 10)		12 W	GDS-15	Significant change in GDS-15 total score from baseline to endpoint for insulin group	N.R.
Hendra [36]	UB RCT	T2DM on oral therapy	Absence	57	Insulin (SCs), twice-daily insulin (*n* = 19), basal/bolus insulin (*n* = 19)	variable dose	Continue oral therapy (*n* = 19)		26 W	HADS	Changes in HADS score was non-significant vs. control group	N.R.
Benedict [37]	DB-RCT	Healthy students	Absence	38	Insulin (IN), (*n* = 19)	160 IU/D	Placebo (*n* = 19)		8 W	EWL-N	Significant difference between insulin and placebo in extroversion, self-confidence, well-being, depression at endpoint	N.R.
Cha [14]	Crossover DB-RCT	Non-diabetes	TR-MDD	35	Insulin (IN)	160 IU/D	Placebo		12 W	MADRS	No between-group differences in change from baseline on total MADRS score or either of the positive or negative subscales of the PANAS	N.R.
Ackermann [38]	DB-RCT	impaired glucose tolerance	Absence	3234	Metformin (*n* = 1073)	1.7 g/D	Placebo (*n* = 1082), intensive lifestyle intervention (*n* = 1079)		52 W	BDI	Small but significant BDI reduction from baseline to endpoint for all three groups	N.R.
Guo [39]	DB-RCT	T2DM	Depression	58	Metformin (*n* = 29)	1–2 g/D	Placebo (*n* = 29)		24 W	MADRS, HDRS-17	MADRS and HDRS-17 scores significantly decreased in metformin group	Depression scores were positively correlated with HbA1c levels
Krysiak [40]	UB, controlled	T2DM or prediabetes	Absence	87	Metformin (*n* = 45)	1.7–3 g/D	TAU (*n* = 42)		26 W	BDI-II	Metformin reduced BDI-II score significantly compared to control	N.R.
Jamilian [41]	DB-RCT	PCOS	Absence	60	Metformin (*n* = 30)	1.5 g/D	Myo-inositol (*n* = 30)	2 g/D	12 W	BDI	Myo-inositol reduced BDI score significantly compared to metformin	N.R.
Rasgon [42]	OL	Non-diabetic IR	MDD or BPD	8	Rosiglitazone (*n* = 8)	8 mg/D	N.A.		12 W	HDRS	Significant declines in HDRS score	Changes in depressive severity scores were not correlated with changes in endocrine variables
Sepanjnia [43]	DB-RCT	Non-diabetes	Moderate to severe MDD	40	Pioglitazone (*n* = 20)	30 mg/D	Placebo (*n* = 20)		6 W	HDRS-17	Pioglitazone group had significantly lower scores at all time points than the placebo group	N.R.
Kemp [21]	OL	Abdominal obesity or MetS	MDD	23	Pioglitazone (*n* = 23)	Average 32.7 mg/D	N.A.		12 W	IDS, QIDS	Pioglitazone significantly reduced IDS and QIDS scores	The reduction in insulin resistance was significantly correlated with improvement in depression severity
Kashani [44]	DB-RCT	PCOS	Mild to moderate MDD (HDRS <20)	40	Pioglitazone (*n* = 20)	30 mg/D	Metformin (*n* = 20)	1.5 g/D	6 W	HDRS-17	Pioglitazone was superior to metformin in reducing HDRS scores at the end of the study	No correlation between changes in HOMA-IR and HDRS
Kemp [45]	OL	MetS or IR	BPD	34	Pioglitazone (*n* = 34)	Average 27.4 mg/D	N.A		8 W	IDS, QIDS	Pioglitazone significantly reduced IDS and QIDS scores	N.R.
Roohafza [46]	DB-RCT	Non-diabetic MetS	Absence	85	Pioglitazone (*n* = 40)	30 mg/D	placebo (*n* = 45)		24 W	HADS-D	Pioglitazone was superior in reducing depression score	Alterations in depression severity were not correlated with changes in insulin resistance level (HOMA-IR)
Lin [47]	DB-RCT	Non-diabetes	Depression	37	Pioglitazone (*n* = 19)	30 mg/D	Placebo (*n* = 18)		12 W	HDRS-21	No significant difference in mean decline in HDRS-21 scores between treatment groups	Within the pioglitazone group, change in HDRS-21 was positively correlated to change in OGTT. Improvement in depression was associated with improvement in glucose metabolism (OGTT) but only in patients with baseline insulin resistance
Hu [48]	UB RCT	T2DM	Post-stroke depression	118	Pioglitazone (*n* = 59)	30 mg/D	Metformin (*n* = 59)	1 g/D	3 M	HDRS-21	HDRS-21 score in the pioglitazone group was lower than that in the metformin group at endpoint	N.R.
Zeinoddini [49]	DB-RCT	Non-diabetes	BPD	44	Pioglitazone (*n* = 22)	30 mg/D	Placebo (*n* = 22)		6 W	HDRS-17	Significantly greater reduction in HDRS scores in the pioglitazone group than in the placebo group	N.R.
Simuni [50]	DB-RCT	Non-diabetes, Parkinson’s disease	Absence	210	Pioglitazone, 15 mg/D (*n* = 72) Pioglitazone, 45 mg/D (*n* = 67)	15 mg/D, 45 mg/D	Placebo (*n* = 71)		44 W	GDS-15	The mean GDS change at 44 w was similar to treatment group	N.R.
Aftab [51]	DB-RCT	Non-diabetes	BPD	37	Pioglitazone (*n* = 17)	15–45 mg/D	Placebo (*n* = 20)		8 W	IDS, MADRS	Borderline significance of *p*-value suggested a strong trend in favor of the placebo (*p* = 0.056) in reducing depressive symptoms (IDS). No significant difference between treatment groups in MADRS score change	No statistically significant correlation between insulin resistance (HOMA-IR) and change in depression score
De Wit [52]	UB RCT	T2DM on insulin	Absence	50	Liraglutide (*n* = 26)	1.8 mg/D	Standard therapy (*n* = 24)		26 W	BDI-II	No significant difference in mean change in BDI-II scores between treatment groups	N.R.
Mansur [33]	OL	Non-diabetes	BP or MDD	19	Liraglutide	1.8 mg/D			4 W	HDRS-17	Liraglutide significantly reduced depressive symptoms	N.R.

BDI: Beck Depression Inventory; BPD: bipolar depression; D: day; DB-RCT: double-blind randomized controlled trial; EWL-N: Eigenschaftswörterliste; GDS: Geriatric Depression Scale; HADS: Hospital Anxiety Depression Scale; HbA1c: Hemoglobin A1c; HDRS: Hamilton Depression Rating Scale; HOMA-IR: homeostatic model assessment for insulin resistance; IDS: Inventory of Depressive Symptomatology; IN: intranasal; IR: insulin resistance; M: months; MADRS: Montgomery Åsberg Depression Rating Scale; MDD: major depressive disorder; MetS: metabolic syndrome; N.A.: not applicable; N.R.: not reported; OGTT: oral glucose tolerance test; OL: open-label; PCOS: polycystic ovarian syndrome; QIDS: Quick Inventory of Depressive Symptoms; SC: subcutaneous; T2DM: type 2 diabetes mellitus; TAU: treatment as usual; UB: unblended; W: weeks.

## Abbreviations

T2DM	Type 2 diabetes
GLP-1RA	Glucagon like peptide-1 receptor agonists
PPAR	Peroxisome proliferator-activated receptor
IR	Insulin receptor
IRS	Insulin receptor substrate
PI3K	Phosphatidylinositol 3-kinase
Akt	Serine/threonine kinase
MAPK	Mitogen-activated protein kinase
ERK	Extracellular signal-regulated kinase
GLP-1	Glucagon-like peptide-1
MDD	Major depressive disorder
GDS	Geriatric Depression Scale
HADS	Hospital Anxiety and Depression Scale
HPA	Hypothalamic–Pituitary–Adrenal
TSST	Trier Social Stress Test
MADRS	Montgomery Åsberg Depression Rating Scale
PANAS	Positive and Negative Affect Schedule
BDI	Beck Depression Inventory
RCT	Randomized clinical trial
HDRS	Hamilton Depression Rating Scale
PCOS	Polycystic ovarian syndrome
MetS	Metabolic syndrome
IDS	Inventory of Depressive Symptomatology
HOMA-IR	Homeostatic model assessment of insulin resistance
QIDS	Quick Inventory of Depressive Symptoms
OR	Odds ratio
CI	Confidence interval
NIRKO	Neuronal-specific knockout of insulin receptor
BCAA	Branched chain amino acids
LAT	Large neutral amino acid transporter
IO and NS	Immune/Inflammatory and Oxidative, and Nitrosative Stress
IL	Interleukin
TNF	Tumor necrosis factor
CRP	C-reactive protein
CNS	central nervous system
IDO	Indolemaine 2,3-dioxygenase
SERT	serotonin transporter
IKKβ	IκB kinase β
NKκB	nuclear factor κB
JNK	c-Jun *N*-terminal kinase
BBB	Blood–Brain barrier
HDL-c	High-density lipoprotein cholesterol
MDA	Malondialdehyde
CAT	Catalase
SOD	Superoxide dismutase
GSH	Glutathione
PON1	Paraoxonase 1
ROS	Reactive oxygen species
NO	Nitric oxide
NOS	Nitric oxide synthase
SAPK	Stress-activated kinases
LTP	Long-term potentiation
LTD	Long-term depression
NMDA	*N*-methyl-d-aspartate
AMPA	a-amino-3-hydroxy-5-methyl-4-isoxazolepropionic acid
IGF	Insulin-like growth factor
BDNF	Brain-derived neurotrophic factor
FOXO	Forkhead box O transcription factor
GC	Glucocorticoid
CRH	Corticotropin releasing hormone
cAMP	Cyclic adenosine monophosphate
PKA	Protein kinase A
STAT	Signal transducer and activator of transcription
GSK	Glycogen synthase kinase
Nrf2	Nuclear factor erythroid 2-related factor 2
COMT	Catechol *O*-methyltransferase
